# Supplementary material for: Reactivity of shape-controlled crystals and metadynamics simulations locate the weak spots of alumina in water
Source: Nat Commun. 2019 Jul 17;10:3139. doi: 10.1038/s41467-019-10981-9 (PMC6637198; doi:10.1038/s41467-019-10981-9)
Supplement: Supplementary file 1 — Supplementary Information [file 41467_2019_10981_MOESM1_ESM.docx]

**Reactivity of shape-controlled crystals and metadynamics simulations**

**locate the weak spots of alumina in water**

Réocreux *et al.*

**Supplementary Information**

**Supplementary Note 1**

**γ-Al_2_O_3_ nanoparticles with different shapes.** All nanoparticles were synthesised from the calcination of boehmite nanoparticles (see details in Methods). The morphology (distribution of fractional facet areas) of the particles was determined from the diffractograms of boehmite before the calcination step since calcination is topotactic and does not modify the shape. Therefore, there is an unequivocal relationship between the shape of boehmite precursors and γ-Al_2_O_3_. Boehmite nanoparticles were considered as lamellar crystals (Figure 1a and Supplementary Figure 1) using a geometry that is frequently observed^1^. The α angle (see definition in Supplementary Figure 1) can be measured from TEM data. Its value is comprised between 95 and 110°, in good agreement with the 104° calculated by Lippens from boehmite lattice parameter^2^. This latter value was then chosen for the calculations. Boehmite diffractograms were deconvoluted in the 2θ ϵ [10°; 70°] domain. In this range of setting, 15 characteristic diffraction lines are indexed in international tables of crystallography (ICDD PDF-2 Database (2016), n°021-1307). The deconvolution was realised using 15 pseudo-Voigt functions positioned on the indexed position of the diffraction lines. A liberty of Δ(2θ) = 0.5° on the left and on the right of the referenced position of the peak was tolerated. Typical dimensions of interest in a boehmite particle are defined in Supplementary Figure 1. Scherrer equation (3) is used to estimate the average thickness *e* and the dimensions *a* and *c* of the boehmite particles. These lengths correspond to the diffraction by the (020), (200) and (002) planes at 2θ ≈ 14.5°, 2θ ≈ 49.2°, and 2θ ≈ 65.0° respectively. *x* and *y* values are then determined using equations (1) and (2). With these lengths, the proportion of each facet area ((010), (001) and (101)) can be easily calculated. Similar method was used by Alphonse *et al.* to provide an estimation of boehmite nanoparticles morphologies^3^. Since the AlOOH/γ-Al_2_O_3_ transition is topotactic, the morphologies of alumina nanoparticles are directly deduced by applying contraction rates^4^ in dimensions x, y and e (Supplementary Table 1). Sizes and shapes of the nanoparticles in alumina P, C and F (resp. plates, commercial and fibres) as determined from XRD analysis (Supplementary Table 2) are in good agreement with the TEM observations (Figure 1g-j). However, this is not the case for alumina R (rods). According to TEM pictures, this alumina should present a very high proportion of lateral (100) facets (*i.e.* dimension y >> x and e) and a small proportion of (111) facets. This difference indicates that boehmite R platelets at the origin of alumina R are most likely polycrystalline. The polycristallinity of such boehmite nanorods has already been observed by Mathieu *et al.*^6^. More generally, the morphologies obtained in the current work are in good agreement with the recent work by Lee *et al.*^5^ that determined the morphologies of boehmite platelets using TEM images.

**Supplementary Figure 1** | Schematics of the top view of a boehmite (AlOOH) nanoparticle. Dimensions of interest in that plane are indexed (see details in text). In addition, the *e* dimension corresponds to the thickness of the particle

**Equations:**

$y=c-\left( \frac{a}{\tan(\pi.\alpha/360)} \right)$ (1)

$x=\sqrt{\left( \frac{c-y}{2} \right)^{2}+\left( \frac{a}{2} \right)^{2}}$ (2)

**Scherrer equation :**

$\varepsilon_{hkl}=\frac{K.k.\lambda}{\sqrt{\left( b_{hkl}^{2}-b^{2} \right).\cos\left( \theta\right)}}$ (3)

*K* is the shape factor of the particle (we chosed 1), *k* is a corrective factor due to the utilization of the full-width half maximum of the peaks, *b*_hkl_ is the full-width half maximum of the (*hkl*) line, *b* is the intrumental width, θ is the (*hkl*) line Braag angle.

**Supplementary Table 1** **|** Unequivocal relationship between facets upon the topotactic calcination of boehmite (AlOOH) into γ-Al_2_O_3_. The variation is given as a contraction rate for each directions^4,7^.

| **Direction** | | **Contraction rate**  **(%)** |
| --- | --- | --- |
| **AlOOH** | **γ-Al_2_O_3_** |  |
| (010) | (110) | 31 |
| (001) | (100) | -6 |
| (101) | (111) | -1.6 |

Relations between γ-Al_2_O_3_ and boehmite (AlOOH) *e*, *x* and *y* dimensions:

$$e_{\mathrm{Al}_{2}O_{3}}=e_{\mathrm{AlOOH}}.0.69$$

$$x_{\mathrm{Al}_{2}O_{3}}=x_{\mathrm{AlOOH}}.1.06$$

$$y_{\mathrm{Al}_{2}O_{3}}=y_{\mathrm{AlOOH}}.1.016$$

**Supplementary Table 2 |** Characterisation of the morphology of the four samples of γ-Al_2_O_3_ consisting of nanoparticles with different edge lengths and facet surface area distributions. S_BET_ is the specific surface area. F, R, P and C respectively stand for fibres, rods, plates and commercial alumina.

|  | **S_BET_ (m²/g)** | $\boldsymbol{e}_{\boldsymbol{Al}_{\boldsymbol{2}}\boldsymbol{O}_{\boldsymbol{3}}}$ **(nm)** | $\boldsymbol{x}_{\boldsymbol{Al}_{\boldsymbol{2}}\boldsymbol{O}_{\boldsymbol{3}}}$ **(nm)** | $\boldsymbol{y}_{\boldsymbol{Al}_{\boldsymbol{2}}\boldsymbol{O}_{\boldsymbol{3}}}$ **(nm)** | **(110) facet**  **(%)** | **(100) facet**  **(%)** | **(111) facet**  **(%)** |
| --- | --- | --- | --- | --- | --- | --- | --- |
|  |  |  |  |  |  |  |  |
| **Alumina F** | 250 | 1.9 | 2.5 | 5.7 | 60 | 21 | 19 |
| **Alumina R** | 179 | 4.8 | 8.4 | 11.5 | 64 | 15 | 22 |
| **Alumina P** | 78 | 16.9 | 21.4 | 6.4 | 50 | 7 | 43 |
| **Alumina C** | 212 | 2.2 | 6.6 | 3.7 | 73 | 9 | 18 |

# Supplementary Note 2

**Adsorption Isotherms.** Supplementary Figure 2 shows adsorption isotherms of sorbitol (a) and xylitol (b) using three aluminas with different morphologies (C, P and F). For all aluminas, the amount of polyol adsorbed increases rapidly with the concentration to a value between 2 and 4 g.L^-1^. At this concentration and for concentrations above, no boehmite was detected by XRD after the hydrothermal treatment and no Al^3+^ was detected in the liquid phase (measured with ICP AES). On the other hand, for concentrations lower than 4 g.L^-1^, alumina dissolution and boehmite precipitation occurred. This is illustrated in Supplementary Figure 3 in the case of xylitol and alumina C: boehmite phase is always detected for concentrations lower than 4 g.L^-1^. The same behaviour was observed in the case of sorbitol and with other alumina. For concentrations higher than 4 g.L^-1^, the amount of polyol adsorbed increases slowlier, indicating that primary adsorption sites become saturated, and that saturation of all those sites is not required to effectively protect alumina from dissolution.

Supplementary Figure 2 shows also that the isotherm profiles are the same for all aluminas but the amount of primary adsorption sites seems to be significantly different. This is confirmed by the fitted Langmuir isotherm parameters (Supplementary Table 3). Indeed, the Langmuir constant is of the same order of magnitude for all aluminas indicating that the adsorption sites involved are the same. The monolayer constant value is however significantly different from one alumina to another. Thus, the number of primary adsorption sites is different from one alumina to the other. We note that after all the treatment performed here, only xylitol or sorbitol was detected in the liquid phase indicating that the polyols did not react under these conditions.

| **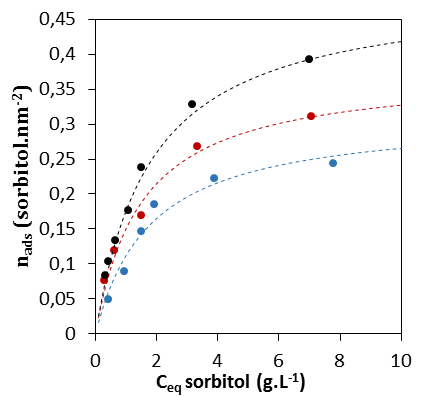**  **(a)** | **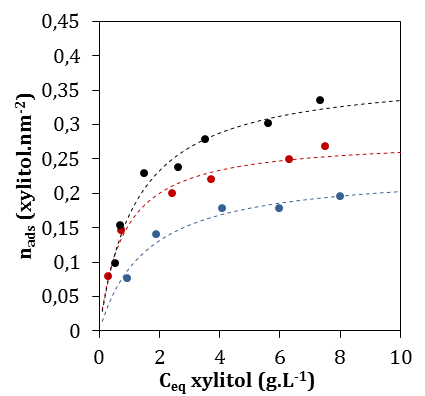**  **(b)** |
| --- | --- |

**Supplementary Figure 2|** Adsorption isotherms at 200°C of sorbitol (**a**) and xylitol (**b**) on alumina C (black), F (red) and P (blue). Dashed lines correspond to the fit with the Langmuir model (see equation (4)). Conditions: 2 g Al_2_O_3_, 50 mL polyol/H_2_O solution, 200°C, autogeneous pressure, 2 h.


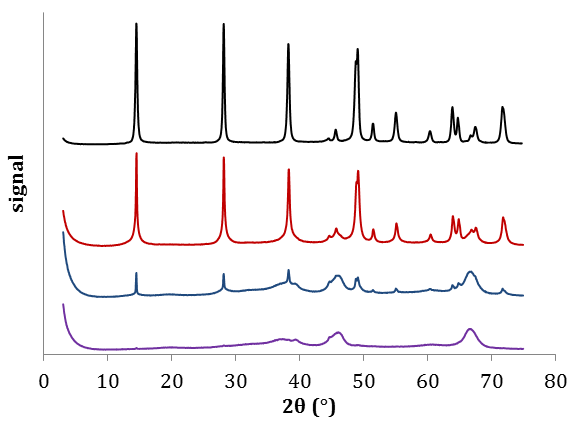


**Supplementary Figure 3** | XRD patterns of alumina C after a 2h hydrothermal treatment at 200°C in aqueous solution of xylitol at 4 g.L^-1^ (purple), 2 g.L-^-1^ (blue), 1 g.L^-1^ (red), and 0 g.L^-1^ (black).

Langmuir equation:

$\frac{n_{\mathrm{ads}}}{n_{\infty}}=\frac{{kC}_{\mathrm{eq}}}{1+{kC}_{\mathrm{eq}}}$ (4)

k is the Langmuir constant (L.mol^-1^), *n*_∞_ is the monolayer constant (molecule.nm^-^²), *n*_ads_ is the amount of polyol adsorbed (molecule.nm^-^²), *C*_eq_ is the equilibrium concentration.

**Supplementary Table 3** | Fitted Langmuir parameters from the experimental curves plotted in Supplementary Figure 2.

|  | **Xylitol** | | | **Sorbitol** | | |
| --- | --- | --- | --- | --- | --- | --- |
| **alumina** | **C** | **F** | **P** | **C** | **F** | **P** |
| ***n*_∞_(molecule.nm^-^²)** | 0.37 | 0.29 | 0.24 | 0.49 | 0.38 | 0.31 |
| ***k* (L.mol^-1^)** | 125 | 181 | 94 | 101 | 121 | 102 |

##

**Supplementary Note 3**

**Inhibition of decomposition and inhibiting coverage.** The inhibition coverage is defined as the amount of polyol adsorbed on the alumina surface at a polyol concentration of 4 g.L^-1^. This is so because all alumina samples were found to require 4 g.L^-1^ as the minimum polyol concentration to reach full protection. This defined concentration was also used for alumina R, without performing any further isotherm analysis. We note that using this concentration of 4 g.L^-1^, no boehmite was detected after a hydrothermal treatment at 200°C for 10 hours (not shown here). In fact, there was no difference between the amount of polyol adsorbed after a high temperature treatment of 2 hours or 10 hours indicating that the adsorption equilibrium is quickly reached.

**Supplementary Table 4 |** Sorbitol and xylitol inhibiting coverages for the four alumina samples used in this study. Inhibiting coverages are determined from the amount of polyol adsorbed during a 200°C hydrothermal treatment in a polyol solution at 4 g.L^-1^. Specific surface areas were determined with N_2_ sorption isotherms at 77 K. The concentration of the remaining polyol in solution was determined by HPLC.

| **Alumina** | **Sorbitol inhibiting coverage**  **(sorbitol.nm^-^²)** | **Xylitol inhibiting coverage**  **(xylitol.nm^-^²)** |
| --- | --- | --- |
| **C** | 0.33 ± 0.02 | 0.28 ± 0.03 |
| **F** | 0.26 ± 0.02 | 0.21 ± 0.03 |
| **P** | 0.22 ± 0.07 | 0.17 ± 0.08 |
| **R** | 0.28 ± 0.03 | 0.23 ± 0.04 |

Under the assumption that both sorbitol and xylitol adsorb specifically to edges, one can also define a lineal inhibiting coverage. To do so, we can first determine the total length D of edges for one particle in each sample using the dimensions given in Supplementary Table 2 (see equation (5)). From these dimensions, one can also determine the surface area of one particle *S*_particle_. The amount *N* of particles in each sample can then be estimated from the BET specific surface area, the mass of the alumina sample m and *S*_particle_ (see equation (6)). Dividing the amount $n_{\mathrm{ads}}$ of polyol adsorbed by the product $N\times D$we obtain the lineal coverage.

$D=8x+6e+4y$ (5)

$N=\frac{S_{\mathrm{BET}}\times m}{S_{\mathrm{particle}}}$ (6)

$\Lambda=\frac{n_{\mathrm{ads}}}{N\times D}$ (7)

**Supplementary Table 5 |** Lineal inhibiting coverage for sorbitol and xylitol for each sample of alumina.

| **Alumina** | **Sorbitol inhibiting coverage**  **(sorbitol.nm^-1^)** | **Xylitol inhibiting coverage**  **(xylitol.nm^-1^)** |
| --- | --- | --- |
| **C** | 1.06 ± 0.08 | 0.90 ± 0.12 |
| **P** | 2.76 ± 0.89 | 1.92 ± 1.30 |
| **R** | 1.41 ± 0.15 | 1.18 ± 0.25 |
| **F** | 0.51 ± 0.04 | 0.42 ± 0.06 |

In Supplementary Tables 4 and 5 errors have been calculated from High Performance Liquid Chromatography (HPLC) measurement of C_eq_ (equation (7)). HPLC standard deviation ($\Delta C)$ value has been determined to be 5% for a concentration range of 0.1 to 1 g.L^-1^ and 2% for the range of 1 to 10 g.L^-1^. Errors due to weight measurement, initial concentration, specific surface area and dimensions of edges have been neglected.

${\Delta n}_{\mathrm{ads}}=n_{\mathrm{ads}}\times\frac{\Delta C}{C_{i}-C_{\mathrm{eq}}}$ (8)

**Supplementary Note 4**

**Relation between inhibiting coverages and the morphology of the nanoparticles.** The total number of adsorbed molecules *N* (say polyol) writes as the sum over the number of adsorbed molecules on each surface:

$N=N_{111}+N_{110}+N_{100}$ (9)

Dividing by the area of the entire surface accessible to adsorbates *S_tot_*, we can write the total coverage:

$\Theta= \frac{N}{S_{tot}}=\frac{N_{(111)}}{S_{tot}}+\frac{N_{(110)}}{S_{tot}}+\frac{N_{(100)}}{S_{tot}}$ (10)

We can then introduce the area of each facets *S_(111)_ , S_(100)_* and *S_(110)_*:

$\Theta= \frac{N}{S_{\mathrm{tot}}}=\frac{S_{(111)}}{S_{\mathrm{tot}}}\frac{N_{(111)}}{S_{(111)}}+\frac{S_{(110)}}{S_{\mathrm{tot}}}\frac{N_{(110)}}{S_{(110)}}+\frac{S_{(100)}}{S_{\mathrm{tot}}}\frac{N_{(100)}}{S_{(100)}}$ (11)

The total coverage simply writes as a sum over the fractional area *x_i_* of each surface times its partial coverage $\theta_{i}$:

$\Theta= \frac{N}{S_{\mathrm{tot}}}=\theta_{\left( 111 \right)}x_{\left( 111 \right)}+\theta_{\left( 110 \right)}x_{\left( 110 \right)}+\theta_{\left( 100 \right)}x_{\left( 100 \right)}$ (12)

In the ideal situation where adsorbates only cover one surface specifically (say the (110) facet), the equation reduces to:

$\Theta= \frac{N}{S_{\mathrm{tot}}}=\theta_{\left( 110 \right)}x_{\left( 110 \right)}$ (13)

Similarly one can derive the same equation assuming that polyols only adsorb on edges. In the particular and ideal case of specific adsorption on one edge *i*, the total lineal inhibiting coverage $\Lambda$ writes as the product of the partial lineal coverage $\lambda_{i}$ on edge *i* with the fraction $x_{i}$ of edge *i*:

$\Lambda=\lambda_{i}x_{i}$ (14)

**Supplementary Note 5**

**Can kinks and edges be involved in the decomposition mechanism of alumina?** First, the amount of kinks is much smaller than the amount of polyol required to inhibit the decomposition of alumina. It is therefore very unlikely that they play a major role in the decomposition mechanism of alumina. As for the edges, the situation is a bit more delicate. In a polydentate configuration, the size of either xylitol or sorbitol is about 1 nm, meaning that lineal coverages cannot go beyond 1 nm^-1^. This corresponds to the order of magnitude of lineal inhibiting coverages determined experimentally and given in Supplementary Table 5. The fact that lineal coverages can experimentally exceed 1 nm^-1^ is already a bit suspicious but could be explained with a certain amount of monodentate species at the edges. We thus need to analyse edges in more details.

To understand the potential role of edges, we have followed the same analysis as that carried out for the facets and described in Figure 1. We assume in this paragraph that the weak spots are on the edges, i.e. decomposition is initiated at the edges and polyols preferably interacts with the edges thereby providing protection of the nanoparticles.

First, it is worth noting that the lineal inhibiting coverages vary between 0.51 and 2.76 nm^-1^ and 0.42 and 1.92 nm^-1^ for sorbitol and xylitol respectively. From one sample to another, only the shape of the nanoparticles changes: the three types of edges (namely E, X and Y see Supplementary Figure 4) are present in different proportions (see Supplementary Table 2). The strong variation of the lineal inhibiting coverages (up to a factor of 5) therefore suggests that adsorption might occur more preferably at one specific edge. When we plot the lineal inhibiting coverage as a function of the fractional length, we see however no correlations (R^2^<0.9). This means that there are no such things as specific interaction with one edge in particular.

The shape effect evidenced by the strong variation of (lineal) inhibiting coverage can seemingly be solely explained for polyol molecules interacting specifically with the (110) facet, as shown in Figure 1.

This is consistent with previous work by Copeland *et al.*^8^ and Larmier *et al.*^9,10^ who were able to explain the properties of moderately hydrated alumina regarding the spectroscopy and reactivity of alcohols/polyols without invoking edges and kinks. It is very likely that the Lewis acid and basic sites at edges and kinks are almost instantaneously saturated with water at the early stage of water adsorption. The resulting aluminol groups are most probably strongly bound to the edges/kinks and cannot be displaced with alcohol/polyol molecules.


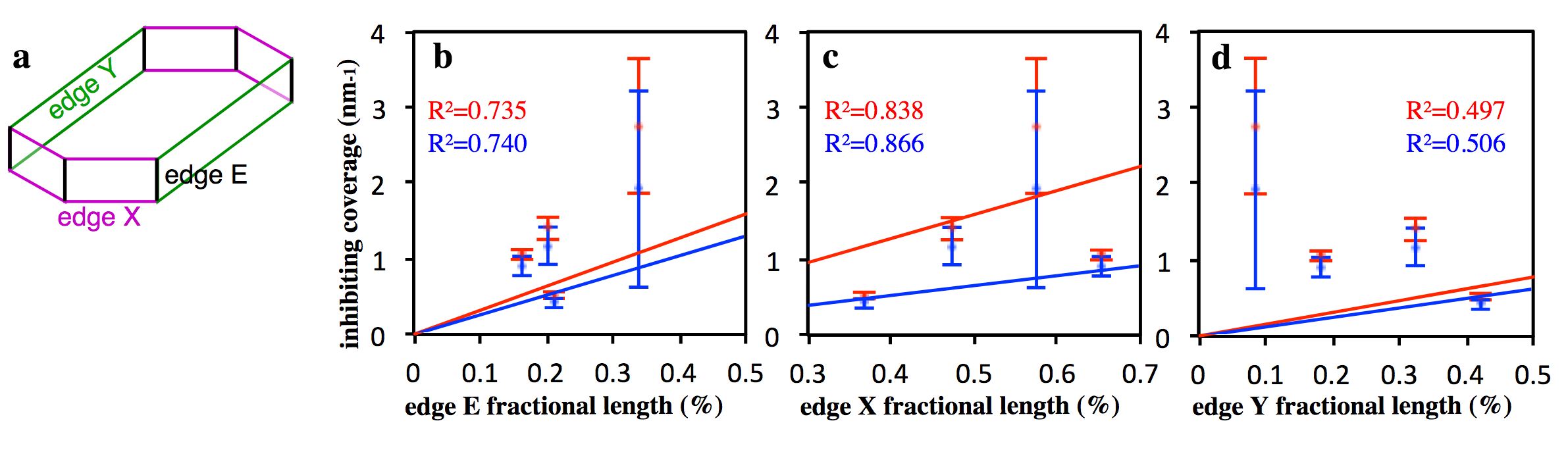


**Supplementary Figure 4 |** Non-implication of edges in the inhibiting adsorption of polyols on the four alumina samples. **a,** General topology of a γ-Al_2_O_3_ nanoparticle exhibiting three major edges: E in black, X in purple and Y in green. **b,c,d**, Comparisons between the *lineal* *inhibiting coverage* (sorbitol in red, xylitol in blue) and the fraction of exposed E (**d**), X (**e**) and Y (**f**) edge lengths. The experimental data sets are fitted to the zero-intercept linear model derived in Supplementary Note 4. The fitted curves are represented as straight lines. No correlation could be established for any edges (R^2^<0.9).

**Supplementary Note 6**

**Theoretical aspects of Metadynamics.** During ab initio molecular dynamics (AIMD), various geometries are sampled as a function of simulation time *t* and can be described using a generalised coordinate *q(t)*. In metadynamics, a bias potential is, on top of that, added to the unknown free energy surface *F(q)* along a few chosen geometric parameters (distance, angles, or any sort of quantities that depend on *q*) called collective variables *s_i_*. For the sake of simplicity, the ensemble of the selected *d* collective variables will be referred to as the vector $\vec{\mathbf{s}}$.

The bias potential is progressively built during AIMD through the periodic addition (stride $\tau$) of small amounts of free energy that takes the form of gaussians. This history-dependent potential $V(\vec{\mathbf{s}},t)$ then writes:

$$V\left( \vec{\mathbf{s}},t \right)=\sum_{k<\frac{t}{\tau}} W\left( k\tau\right)\exp\left( -\sum_{i} \frac{\left( s_{i}-s_{i}\left( q\left( k\tau\right) \right) \right)^{2}}{2\sigma_{i}^{2}} \right)$$

where *W* is the height of the Gaussians and $\sigma_{i}$ the width along the direction of collective variable *s_i_*. For standard metadynamics *W* is a constant, but for well-tempered metadynamics, *W* decreases as a function of the amount of Gaussians previously added locally:

$W\left( k\tau\right)=W_{0}\exp\left( -\frac{V(\vec{\mathbf{s}}(q\left( k\tau\right),k\tau)}{k_{B}\Delta T} \right)$ (15)

The decay is controlled using the parameter $\Delta T$. This procedure allows for a smoothlier convergence of the bias potential. In the limit of long simulation times, the bias potential converges and is related to the free energy surface projected along the selected collective variables.

$V\left( \vec{\mathbf{s}},t\to\infty\right)= -\frac{\Delta T}{T+\Delta T}F\left( \vec{\mathbf{s}} \right)+\text{constant}$ (16)

Practically, we do not choose directly $\Delta T$ when using Plumed/CP2K but rather the bias factor $\gamma$:

$\gamma=\frac{T+\Delta T}{T}$ (17)

The bias factor was set to 100. The details of the set-up (collective variables and parameters) of the metadynamics simulations are given in the Methods section in the main article.

The collective variables we chosen are based on coordination numbers. To describe the coordination of aluminium Al_j_ with a set of oxygens {O_set_} CN is defined by:

$CN(Al_{j},O_{set})= \sum_{i\in\{O_{set}\}} s_{ij}(r_{ij})= \frac{1-\left( \frac{r_{ij}-d_{0}}{r_{0}} \right)^{n}}{1-\left( \frac{r_{ij}-d_{0}}{r_{0}} \right)^{m}}$ (18)

with *r_ij_* the inter-atomic distance between atom i and atom j, *s_ij_*(*r_ij_*) the switching function describing the coordination between atom *i* and *j*, *d*_0_ the central value of the switching function (*s_ij_*(*d*_0_) = 1), *r*_0_ the acceptance distance of the switching function, and with *n* and *m* two integer exponents with *n* < *m*. The switching function *s_ij_* is plotted for a given value of *r*_0_ and various ratios *n*/*m* to illustrate the effect of those parameters on its shape in Supplementary Figure 5. *d*_0_ is chosen to match the Al-O equilibrium distance (1.5Å). *r*_0_ can be seen as an acceptance distance which, coupled with the *n*/*m* ratio, controls at which distance the O atom is not anymore considered as bonded to Al. At *d*_0_+*r*_0_, the switching function *s_ij_* is equal to *n*/*m*. The *n*/*m* ratio can be seen as the swiftness of decrease of the function away from the equilibrium distance *d*_0_. Here, a ratio of 2/5 (*n*=4, *m*=10) and a *r*_0_ of 0.9Å has been chosen and we can, therefore, observe that this switching function has a value of about zero for an interatomic distance greater than 3Å.

**Supplementary Figure 5** | Switching function *s_ij_* for *r*_0_ =0.9 Å and *n*/*m* ratio with *n*=4. The corresponding mathematical definition is given in the text.

**Supplementary Note 7**

**Reactivity of Al_α_ sampled with metadynamics**. Performing the metadynamics simulation on Al_α_, we reconstructed the free energy surface projected on CN_a_ and CN_w_, the coordination of Al_α_ to the oxygen atoms of alumina and water respectively, as shown on Supplementary Figure 6. Within a free energy span of about 200 kJ.mol^-1^, no other minimum than the one corresponding to the initial structure at (3,1) can be defined. This shows that Al_α_ is rather unreactive towards water.

**Supplementary Figure 6** | Free energy surface of Al_α_ computed from the metadynamics simulation using two collective variables, CN_a_ that represents the Al coordination to alumina oxygen atoms and CN_w_ the one to water oxygen atoms.

**Supplementary Note 8**

**Sites of adsorption.** The *ab initio* molecular dynamics (AIMD) simulations were performed as detailed in the Methods section of the main article. We used, as a reference, our recently published AIMD simulation on the γ-Al_2_O_3_(110)/water interface^11^, with the same simulation set-up. Starting from this equilibrated interface, we substituted 5 water molecules (2 chemisorbed H_2_O, 1 chemisorbed OH and 2 physisorbed water molecules) with xylitol as illustrated in Supplementary Figure 7. We considered three different tridentate geometries. The choice of these tridentate geometries is motivated by both literature and the present work:

1. We show in the Supplementary Note 3 that sorbitol and xylitol reach a saturation of adsorption for about 1/4 to 1/3 of a monolayer. If the interaction with the surface was driven by non-specific interaction, we should in principle be able to saturate the surface. This low coverage saturation thus suggests that the adsorption relies on specific interaction rather than non-specific physisorption. The corresponding free energy of adsorption is moderate for chemisorption (-20 kJ.mol^-1^ according to our Langmuir constant) but is consistent with a substitution of water molecules with alcohol moieties on the surface.

**Supplementary Figure 7 |** Adsorption of glycerol and xylitol through the substitution of water molecules. **a,** Structure of γ-Al_2_O_3_(110) surface saturated with water molecules. **b,** Substitution of chemisorbed water molecules with glycerol (structure proposed by Copeland *et. al.*^8^) and xylitol (geometry proposed in the present work) and various views of a snapshot of xylitol at the Al_2_O_3_-water interface (O in red except O from water in blue, Al in yellow, C in black and H in white).

1. The only polyols for which the adsorption geometry on γ-Al_2_O_3_(110) has been studied in the literature is glycerol, which happens to adsorb with a bidentate geometry^8,12^. We can infer that xylitol and sorbitol would also have a multi-dentate adsorption mode. Considering the local geometry of γ-Al_2_O_3_ (110), it is possible to propose a tridentate adsorption geometry for those C5 and C6 polyols. Moreover, it is worth noting that glycerol is also an inhibitor of the decomposition of γ-Al_2_O_3_^13^. Since all three are polyols, we can assume that they inhibit the decomposition via similar mechanisms. Glycerol however is only able to slow down the decomposition, whereas sorbitol and xylitol totally inhibit the decomposition. The latter must therefore interact more with the surface, which is consistent with a tridentate geometry.
2. The present work shows the importance of chemisorbed water molecules in the decomposition mechanism of γ-Al_2_O_3_ in liquid water. With its partial substitution of water molecule on the surface, glycerol leaves some water molecules behind that can potentially react with the leaching Al atoms. With a tridentate adsorption mode, the probability of having such chemisorbed water molecules in the vicinity of the particularly water-sensitive Al centre vanishes.

**
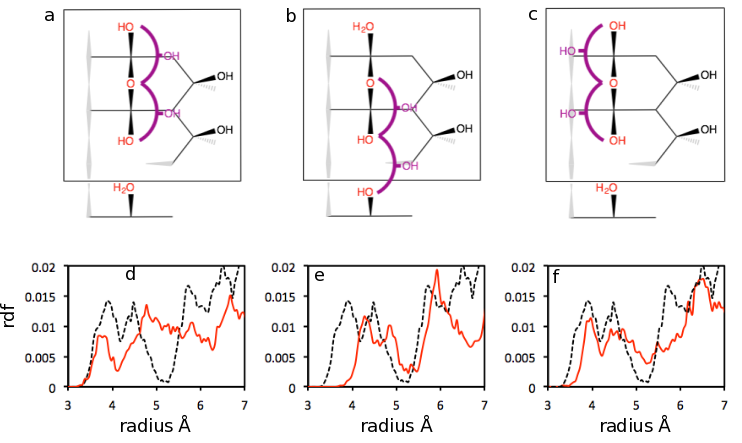
**

**Supplementary Figure 8 |** γ-Al_2_O_3_(111)/water interface including adsorbed xylitol in three different geometries (**a**, **b** and **c**). The radial distribution of Al_β_ with free water molecules is given in absence (dash black) and in presence (solid red) of xylitol to show the influence of its adsorption on the structuration of the interface depending on the adsorption mode (**d**, **e** and **f**).

We performed one AIMD simulation for each adsorption mode of xylitol. After 10 ps of thermalisation, we ran the simulations for 25 ps for production. We compared the structuration of the free water molecules around the water-sensitive Al_β_ with the reference interface using radial distribution functions (see Supplementary Figure 8). When the two OH moieties of xylitol point toward the tetrahedral Al atoms (Supplementary Figure 8a-b), the structuration of free water molecules is highly impacted: the second coordination shell (the first being the chemisorbed OH group on Al_β_) either has a smaller concentration (Supplementary Figure 8d) or is pushed further to larger radii (Supplementary Figure 8e). The OH group indeed induce bulk hindrance that makes those second coordination shell water molecules less accessible to Al_β_. For the last geometry (Supplementary Figure 8c) with the OH moieties opposite to the tetrahedral centres, the effect can also be detected, albeit to a lesser extent.

To better understand the protecting effect of the polyol, we determined if this is related to the absence of water in the neighbourhood of the Al_β_ centre. Firstly, the water density is determined on a fine grid from the accumulation of the 30 ps production run of molecular dynamics using the mean occupied volume of each atom from its van der Waals radius. In Figure 3, the iso-surface represents an iso-value of this water density. This iso-value has been chosen to nicely separate well-occupied zones and poorly occupied zones and has a value of 2. This value of 2 means that, each point of this iso-surface of water density is overlapping with 2 van der Waals atoms on average over time.

**Supplementary Note 9**

**Reactivity of Al_β_ sampled with metadynamics in presence of adsorbed xylitol**. The metadynamics simulation corresponding to the first adsorption mode of xylitol (Supplementary Figure 8a) is detailed in the main article. As for the second and third adsorption geometries, the reconstructed free energy surfaces are given in Supplementary Figure 9a and 9b respectively.

Supplementary Figure 9 | Free energy surface obtained from the metadynamics simulation on Al_β_ in presence of xylitol adsorbed as in Supplementary Figure 8b (a) and Supplementary Figure 8c (b). CN_a_ is the coordination number of Al_β_ to alumina oxygen atoms. CN_w+p_ is the coordination number of Al_β_ to water and polyol oxygen atoms.

We have already mentioned in Supplementary Note 7 that the adsorption geometries a and b for xylitol show a similar influence on the structure of water molecules around Al_β_ (second coordination shell). This results in an analogous reactivity towards water as shown by the very similar free energy surfaces displayed in Figure 3 (main article) and Supplementary Figure 9a. Second coordination shell water molecules cannot access Al_β_ and the mechanism therefore switches from a two-step addition and elimination mechanism to a S_N_2 mechanism with a strong S_N_1 character. Al_β_ reacts with xylitol instead of water, upon the de-coordination from one oxygen atom belonging to alumina. There is therefore no need for extra physisorbed water molecule to come to saturate the octahedral structure of the product.

In the last structure (Supplementary Figure 8c), which has the two uncoordinated alcohol moieties opposite to the tetrahedral centres, the reactivity is different (Supplementary Figure 9b). As expected, xylitol cannot chelate the leaching Al_β_ in the same manner. Al_β_ is able to de-coordinate from one oxygen atom of alumina and to coordinate with one alcohol moiety of xylitol (product at (2,2)) through a S_N_2 mechanism. The barrier is higher than in absence of xylitol (95 kJ.mol^-1^ to compare with 78 kJ.mol^-1^ in absence of xylitol). In addition, no other minimum can be observed for a span of free energies of 180 kJ.mol^-1^, probably because of the constraint and hindrance induced by the C-H bonds of xylitol.

In short, all three adsorption modes show that physisorbed water molecules cannot react anymore with Al_β_ directly. The only reactivity observed is limited with that involving xylitol and does not allow the dissolution of Al centres.

# Supplementary References

1. Busca, G. The surface of transitional aluminas: A critical review. *Catal. Today* **226,** 2–13 (2014).

2. Abi Aad, J. *et al.* Chemical weathering of alumina in aqueous suspension at ambient pressure : a mechanistic study. *ChemCatChem* **9,** 2186–2194 (2017).

3. Alphonse, P. & Courty, M. Structure and thermal behavior of nanocrystalline boehmite. *Thermochim. Acta* **425,** 75–89 (2005).

4. Lippens, B. C. Structure and Texture of Aluminas. (Technische Hogeschool of Delft, The Netherlands, 1961).

5. Lee, J., Jang, E. J., Jeong, H. Y. & Kwak, J. H. Critical role of (100) facets on γ-Al2O3 for ethanol dehydration: Combined efforts of morphology-controlled synthesis and TEM study. *Appl. Catal. A Gen.* **556,** 121–128 (2018).

6. Mathieu, Y., Lebeau, B. & Valtchev, V. Control of the morphology and particle size of boehmite nanoparticles synthesized under hydrothermal conditions. *Langmuir* **23,** 9435–9442 (2007).

7. Digne, M., Sautet, P., Raybaud, P., Euzen, P. & Toulhoat, H. Use of DFT to achieve a rational understanding of acid-basic properties of γ-alumina surfaces. *J. Catal.* **226,** 54–68 (2004).

8. Copeland, J. R., Shi, X.-R., Sholl, D. S. & Sievers, C. Surface Interactions of C2 and C3 Polyols with γ-Al2O3 and the Role of Coadsorbed Water. *Langmuir* **29,** 581–593 (2013).

9. Larmier, K. *et al.* Influence of Coadsorbed Water and Alcohol Molecules on Isopropyl Alcohol Dehydration on γ-Alumina: Multiscale Modeling of Experimental Kinetic Profiles. *ACS Catal.* **6,** 1905–1920 (2016).

10. Larmier, K. *et al.* Mechanistic Investigation of Isopropanol Conversion on Alumina Catalysts: Location of Active Sites for Alkene/Ether Production. *ACS Catal.* **5,** 4423–4437 (2015).

11. Réocreux, R., Jiang, T., Iannuzzi, M., Michel, C. & Sautet, P. Structuration and Dynamics of Interfacial Liquid Water at Hydrated γ-Alumina Determined by ab Initio Molecular Simulations: Implications for Nanoparticle Stability. *ACS Appl. Nano Mater.* **1,** 191–199 (2018).

12. Copeland, J. R., Santillan, I. A., Schimming, S. M., Ewbank, J. L. & Sievers, C. Surface Interactions of Glycerol with Acidic and Basic Metal Oxides. *J. Phys. Chem. C* **117,** 21413–21425 (2013).

13. Ravenelle, R. M., Copeland, J. R., Van Pelt, A. H., Crittenden, J. C. & Sievers, C. Stability of Pt/γ-Al2O3 Catalysts in Model Biomass Solutions. *Top. Catal.* **55,** 162–174 (2012).
